# Application of Air-Coupled Ultrasonic Arrays for Excitation of a Slow Antisymmetric Lamb Wave

**DOI:** 10.3390/s18082636

**Published:** 2018-08-11

**Authors:** Rymantas J. Kazys, Almantas Vilpisauskas, Justina Sestoke

**Affiliations:** Ultrasound Institute, Kaunas University of Technology, LT-51423 Kaunas, Lithuania; almvilp@gmail.com (A.V.); justina.sestoke@ktu.lt (J.S.)

**Keywords:** air-coupled ultrasonic arrays, ultrasonic guided waves, lamb waves, non-destructive testing, evaluation

## Abstract

Air-coupled excitation and reception of ultrasonic guided waves is already used for non-destructive testing and evaluation (NDT & E). Usually for air-coupled NDT & E purposes the lowest zero-order antisymmetric Lamb wave mode A_0_ is used, because it is most sensitive to internal defects and thickness variations. The velocity of the A_0_ mode is reduced with a reducing frequency and at low frequencies may become slower than the ultrasound velocity in air. Such a wave is named a slow Lamb wave. The objective of this research was the development and investigation of an air-coupled excitation method of the slow zero-order antisymmetric Lamb wave based on application of a piezoceramic ultrasonic array. We have proposed to excite the A_0_ mode by a planar air-coupled phased array with rectangular elements. The array is matched to the wavelength of the A_0_ mode in the film. Performance of such an excitation method was investigated both theoretically and experimentally. Two excitation methods of the array were analysed: when all array elements were excited simultaneously or one by one with a proper delay. In order to reduce crosstalk between array elements via the air gap, we have proposed an optimization procedure based on additional shifts of electric excitation impulses of the array elements. For experimental verification of the proposed approach a prototype of the air-coupled eight element array made of Pz-29 piezoceramic strips was manufactured. Experimental validation confirmed the possibility of exciting the slow A_0_ Lamb wave mode through the air gap in thin plates and films.

## 1. Introduction

Lamb waves (or guided waves) are already used for non-destructive testing and evaluation (NDT & E) of sheet type materials. Usually such waves are excited by an ultrasonic transducer coupled to the structure under a test by a coupling liquid. However, there are cases when such approach is not suitable, for example, because the tested material is hot, or may be damaged or contaminated by a coupling liquid. Typical examples of such materials are paper, wood and some plastic and aerospace components.

In this case various non-contact excitation methods of the Lamb waves are used. For this purpose, different techniques are applied, for example excitation by lasers [1,2], electrostatic excitation methods [2], or electromagnetic acoustic transducers [3,4]. Some of them are suitable only for electrically conductive materials, therefore air-coupled excitation and reception of guided waves is already used for NDT of plate and rod-type composite materials or measurement of material properties [5,6,7,8,9,10,11,12,13,14,15]. The popularity of this method is still increasing, but broader usage is limited by the significant insertion losses of ultrasonic signals, which may reach up to 120–160 dB. Attenuation of acoustic waves in air and materials under test usually increases non-linearly together with the frequency, therefore ultrasonic waves of lower frequencies (<50 kHz) are often used [16,17]. Application of such waves enables one to implement so called long range ultrasonic testing that allows fast (~several minutes) in situ NDT of plate structures with large dimensions [12].

There is a special class of widely used very thin (~0.1 mm) materials—plastic tapes and films like thermoplastic CFRP tapes [18] and clear polyvinyl chloride (PVC) films. For example, production of PVC films in Europe reaches 5 million tons annually [19]. Such thin tapes and films are flexible and unstable therefore they may be efficiently inspected on-line only using air-coupled ultrasonic methods [18].

Usually for air-coupled NDT purposes the lowest zero-order antisymmetric Lamb wave mode A_0_ is used because it is most sensitive to internal defects and thickness variations. For very thin free isotropic homogeneous plates and low frequencies the phase velocity *c_A_*_0_ of the A_0_ mode is given by the following equation [20]:

(1) $$c_{A0}^{2}=\sqrt{\frac{E}{12\rho \left(1-\nu^{2}\right)}}2\pi fd$$

where *E* is the Young’s modulus, *ρ* is the density, *ν* is the Poisson’s ratio, *f* is the frequency and *d* is the thickness of the plate. When the phase velocity in a plate is higher than the ultrasound velocity in the air, the propagating A_0_ mode radiates a leaky wave into air. Such Lamb waves are usually exploited for detection of defects in tested materials, because the leakage field increases strongly in the defect zone [14]. From Equation (1) follows that the velocity of the A_0_ mode is reduced with the reduced frequency and at low frequencies may become close or even lower than the ultrasound velocity in air. Then the Lamb wave is trapped in the tested material and no leaky waves exist. Such a wave we shall name a slow Lamb wave. Propagation of the slow ultrasonic wave in a plate is accompanied by a non-propagating evanescent wave in air. Application of such waves may be attractive for NDT of thin flexibles plates and films because due to lower losses such waves might propagate longer distances. As it will be shown in this paper, conventional excitation methods of slow A_0_ Lamb wave mode using a deflected incident ultrasonic plane wave are not feasible. This problem may be overcome exploiting for this purpose air-coupled ultrasonic array matched to the wavelength of the Lamb wave in a plate under a test.

Excitation of Lamb waves by an ultrasonic array may look similar to excitation of surface acoustic waves in solid substrates, however in the case of air-coupled arrays there are essential differences. First of all, due to losses in air and properties of composite or polymer materials the operation frequency is much lower. The wavelength of an ultrasonic wave in air is comparable with a width of the array elements what results in diffraction effects in air and complicates excitation of required modes of the Lamb waves. The objective of this research was development and investigation of an air-coupled excitation method for slow zero-order antisymmetric Lamb wave mode based on application of an ultrasonic array.

## 2. Air-Coupled Excitation Principle and Problems

**Figure 1:**
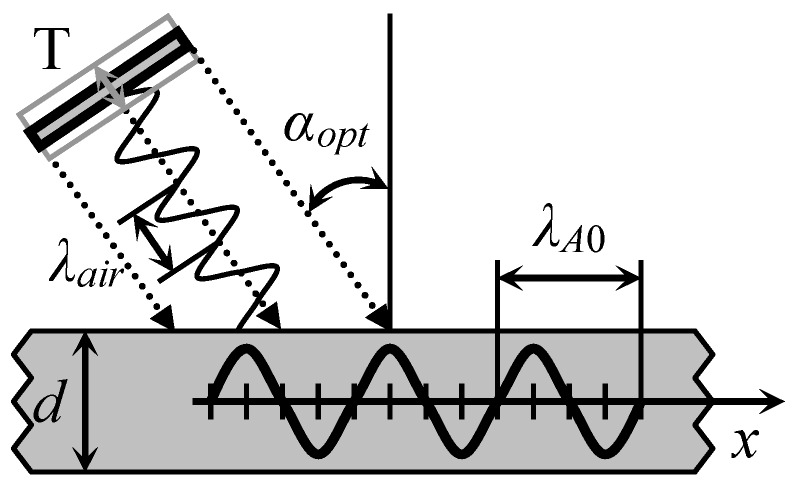
Principle of air-coupled Lamb wave excitation according to the Snell’s law.

Usually for air-coupled excitation and reception of the antisymmetric Lamb wave A_0_ mode ultrasonic transducers are oriented with respect to the plate structure under a test at the optimum angle *α_opt_* (Figure 1).

In the case of a plane wave the value of this angle *α_opt_* is found from the Snell’s law:

(2) $$\alpha_{opt}=\arcsin\frac{\lambda_{air}}{\lambda_{A0}\left(f\right)}=\arcsin\frac{c_{air}}{c_{A0}\left(f\right)}$$

where *λ_air_* and *c_air_* are respectively the wavelength and the sound velocity in air; *λ_A_*_0_(*f*) and *c_A_*_0_(*f*) are the frequency dependent wavelength and phase velocity of the A_0_ Lamb wave mode in the structure under a test.

**Figure 2:**
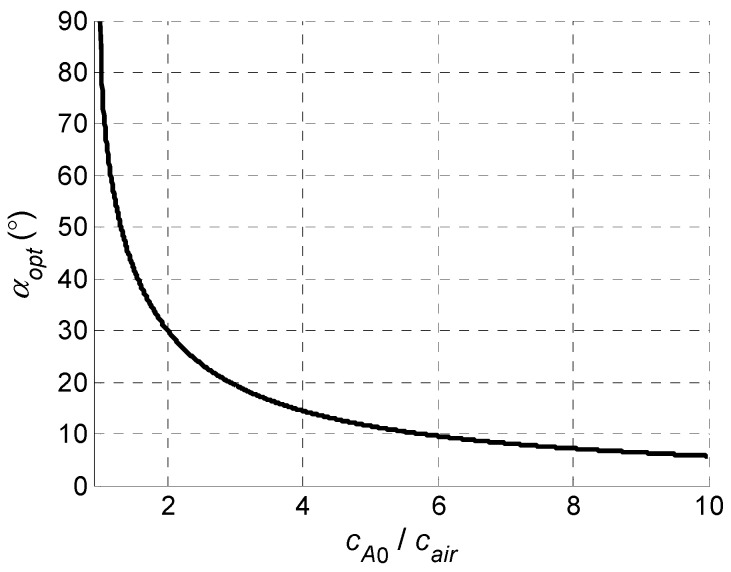
Dependence of the optimum excitation angle *α_opt_* when the phase velocity *c_A_*_0_ in an object under a test is greater than the sound velocity in air *c_air_*.

The value of the optimum incidence angle *α_opt_* according to the Snell’s law (Equation (2)) depends on the ratio of the phase velocities *c_air_*/*c_A_*_0_(*f*). For the case when the ultrasound phase velocity in the structure is greater or equal than the sound velocity in air *c_A_*_0_(*f*) ≥ *c_air_*, the optimum incidence angle *α_opt_* calculated according to Equation (2) is presented in Figure 2.

When the ultrasound velocity in a plate *c_A_*_0_ is less than 2*c_air_*, the value of the optimum excitation angle *α_opt_* begins to grow very quickly and at lower velocities cannot be realized due to the finite dimensions of the ultrasonic transducer.

**Figure 3:**
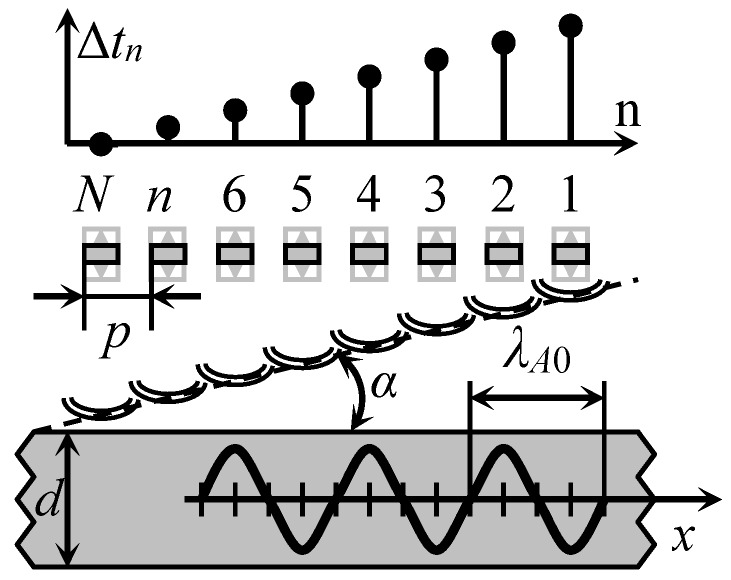
Air-coupled excitation of the A_0_ Lamb wave mode by a phased array with rectangular elements.

In order to overcome this problem, we have proposed to excite the A_0_ mode by a planar air-coupled phased array with rectangular elements [21]. In this case the array aperture is parallel to the surface of the plate or film type material, as shown in Figure 3.

By introducing suitable delays ∆*t_n_* between the excitation instants of the array elements it is possible get an electronically controlled deflection angle α of the incident ultrasonic wave.

The optimum incidence angle α*_opt_*, necessary for excitation of the A_0_ mode, can be obtained by introducing time delays between electric excitation signals. The delay times Δ*t_n_* for each array element are calculated according to:

(3) $$\Delta t_{n}=p\left(N-n\right)\frac{\sin\alpha_{opt}}{c_{air}}$$

where *p* is the array pitch; *N* is the number of elements in array; *n* is the element number. As it was mentioned above, the angle *α_opt_* depends on the ratio of ultrasound velocities in air and in the film *c_air_*/*c_A_*_0_(*f*) and is frequency dependent.

The values of the A_0_ Lamb wave mode phase velocity in PVC films of different thickness were calculated using the Semi Analytical Finite Element (SAFE) method. For calculations the parameters of the clear PVC film presented in Table 1 were used [22].

**Figure 4:**
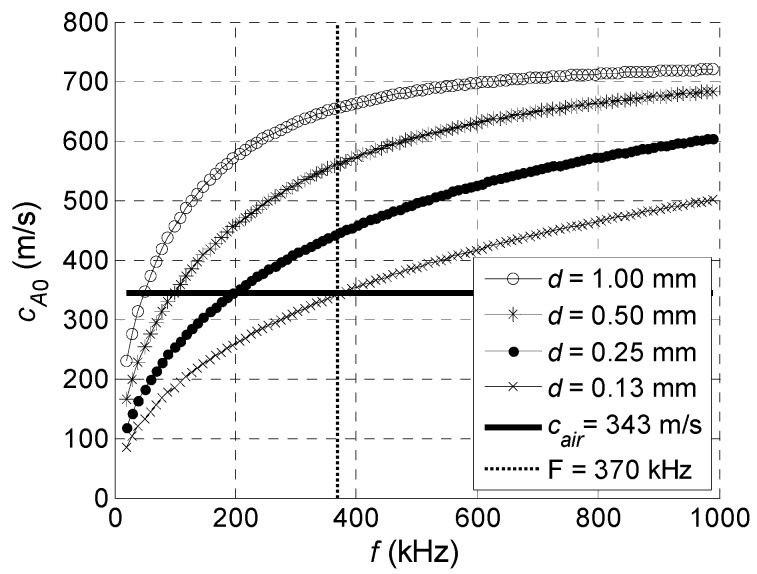
Calculated phase velocities *c_A_*_0_ of the A_0_ Lamb wave mode in PVC films of different thickness *d*.

The results are shown in Figure 4. The ultrasound velocity in air *c_air_* = 343 m/s is indicated by a solid horizontal line.

As it follows from the results presented, the phase velocity *c_A_*_0_ decreases with decreasing frequency and decreasing thickness of the film. At lower frequencies (<370 kHz, vertical dotted line), depending on the thickness of the film, the phase velocity in the PVC film becomes lower than the ultrasound velocity in air. Referring to the calculated optimum incidence angles presented in Figure 2, it looks that in this region it would be impossible to excite the A_0_ mode.

**Figure 5:**
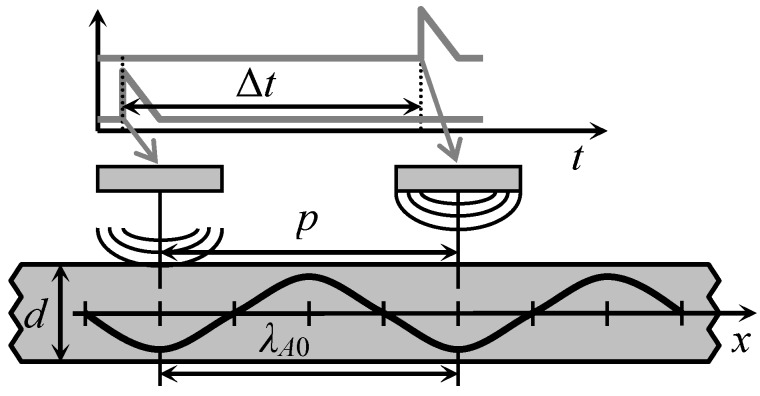
Principle of air-coupled A_0_ Lamb wave mode excitation using phased array with rectangular elements, when the phase velocity is lower than ultrasound velocity in air.

This problem may be overcome by using the air-coupled linear array, matched to the wavelength of the A_0_ mode in the film. This kind of matching is achieved by selecting the pitch—the distance *p* between the centres of neighbouring array elements equal to the wavelength of the A_0_ mode *p* = *λ_A_*_0_ at the selected frequency (Figure 5).

The width of the array elements must be less than λ*_A_*_0_/2. In this case each array element is producing excitation in-phase with the guided wave propagating under array.

**Figure 6:**
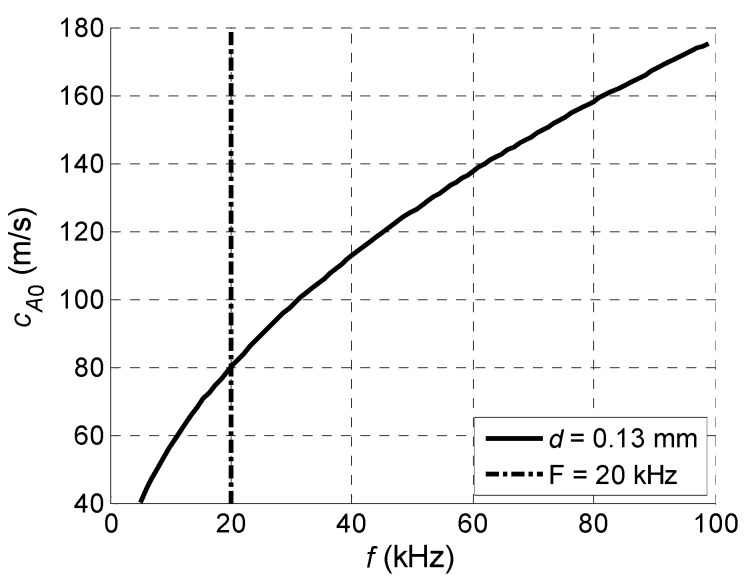
A_0_ mode phase velocity *c_A_*_0_ dispersion curve for the PVC film with *d* = 0.13 mm thickness.

**Figure 7:**
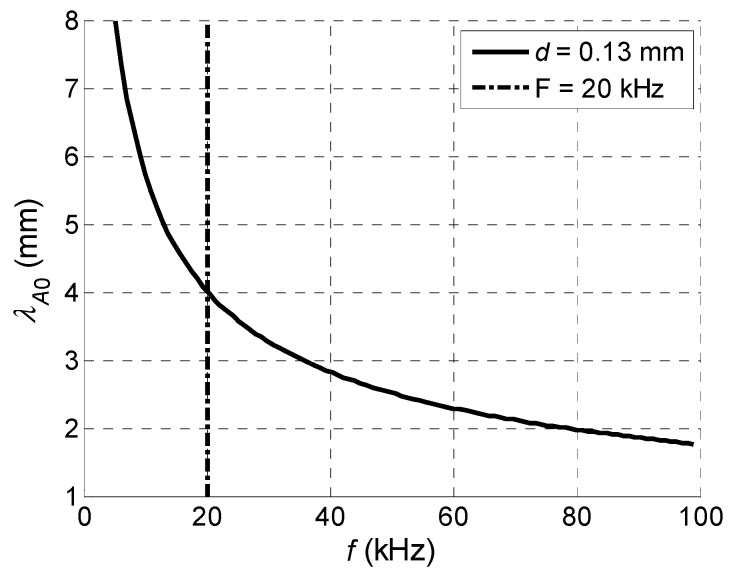
A_0_ mode wavelength *λ_A_*_0_ versus frequency *f* for PVC film with *d* = 0.13 mm thickness.

Let us analyse if an array with such requirements may be realized. The main limiting factor now is the frequency dependent wavelength of the A_0_ mode. The zoomed phase velocity dependence in the PVC film of 0.13 mm thickness versus frequency is shown in Figure 6 and the corresponding wavelength in Figure 7.

From the results presented it follows that with a reduced frequency, in spite of the reduced A_0_ mode velocity the wavelength is increasing and is on the order of a few millimetres. For example, at the *f* = 20 kHz frequency indicated by a vertical dot-dashed line in Figure 7, the wavelength λ*_A_*_0_ = 4 mm. As it will be shown below, the array with such pitch can be realized using strip like piezo ceramic elements, vibrating in a thickness extension mode.

The delay time step between two array elements Δ*t* must be equal to the time, required to travel the distance *p* and is given by:

(4) $$\Delta t=\frac{p}{c_{A0}\left(f\right)}=\frac{\lambda_{A0}}{c_{A0}\left(f\right)}$$

From Equation (4) follows that by selecting delay times of the excitation pulses of different array elements it is possible to excite the waves the wavelength of which is not strictly equal to the pitch between elements. This also allows compensating variances of the pitches caused by any inaccuracy occurring during manufacturing process of the array. Finally, selection of the proper delay times allows adapting such array to films and thin sheets with different ultrasound velocities.

## 3. Theoretical Analysis

Performance of the proposed excitation method was investigated by modelling, which includes propagation of an ultrasonic wave through the air gap between the array and the film and excitation of a guided wave in the film. The acoustic pressure generated by each array element with a rectangular aperture was calculated using the impulse response method (IRM) [23,24,25,26]. Such approach takes into account diffraction effects which are clearly expressed in the near field zone of the array. A pressure impulse propagates through the air, meets the surface of plate and generates Lamb waves. If an infinite isotropic plate is excited by an incident time harmonic signal, then normal displacements in the plate can be calculated using the time harmonic solution (THS) method [27].

The above described IRM and THS methods were realized in a free software tool “The Lamb Matlab toolbox” (Beta version 0.1) [27]. In this software package a finite excitation zone on a surface of the film is divided into circular sub-regions of radius *a*. The pressure signal is calculated at the centre point of each circular sub-region and is taken the same in the whole sub-region area. The radius *a* is chosen small, for example, at least four times shorter than the minimum Lamb wavelength. The total off-plane normal displacements at the given point on a plate are obtained by superposition of all normal displacements, created by all circular sub-regions.

**Figure 8:**
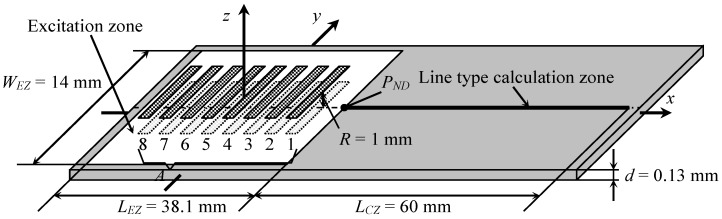
Schematic diagram of air-coupled Lamb wave excitation using planar phased array A.

Numerical investigation was performed simulating an air-coupled A_0_ Lamb wave mode excitation by a planar linear array A (Figure 8).

In order to obtain similar theoretical and experimental results, parameters of the modelled array A were chosen according to the manufactured piezoelectric array, used in experiments (see experimental investigations section for more details). It consists of eight narrow rectangular elements with dimensions of 1 mm × 7 mm. All spacing’s between elements should theoretically be equal to 3.3 mm, but in the manufactured array they are slightly different. Coordinates of the array elements centres along *x*-axis are given in Table 2, so the theoretical values *X_T_* and the real values *X_R_* can be compared.

As it follows from the presented data, the differences between theoretically required and actually achieved distances between array elements are quite small. However, in order to get a reliable modelling result we have modified the software “The Lamb Matlab toolbox”, because it does not allow simulating array with different distances between array elements.

The structure of the acoustic pressure field radiated by each array element changes along the distance *z*. The near field zone limit *L_NF_* for a rectangular aperture with dimensions 2*a* × 2*b* is given by [28]:

(5) $$L_{NF}=\frac{2a\cdot 2b}{\pi \cdot \lambda_{air}}$$

**Figure 9:**
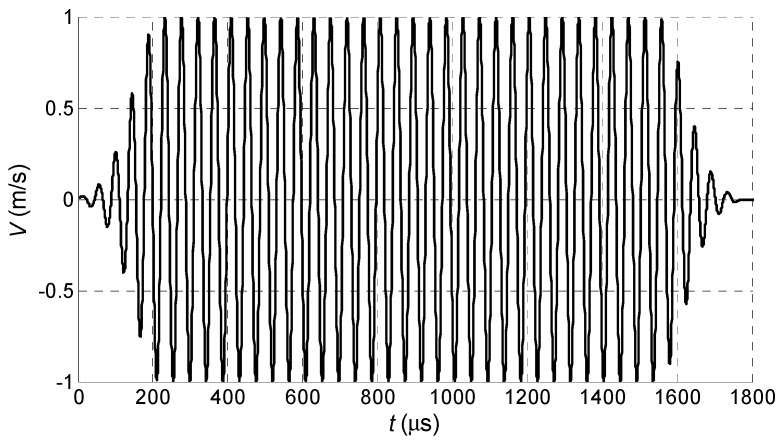
Particle velocity impulse *V* radiated by an array element: the main frequency 22.62 kHz.

For the element with dimensions 2*a* = 1 mm and 2*b* = 7 mm, the near field zone is *L_NF_* = 0.15 mm. The array A was located at *R* = 1 mm distance from the PVC film, what means that the film is in a far field zone. It was assumed that elements of the array A radiate a particle velocity signal *V* with the main frequency 22.62 kHz (this is the value that worked best in experiments), maximum amplitude 1 m/s and duration 40 periods. In order to avoid digital artefacts during simulations the half Gauss window function was applied at the beginning and at the end of the excitation signals (Figure 9).

The pressure signal acting on the surface of the film is calculated within a selected finite rectangular excitation zone. The most significant pressure values are created under the array A in a rectangular projection zone with dimensions of 31.1 mm × 7 mm. In order to achieve accurate simulation results, the dimensions of the rectangular excitation zone are selected bigger with an additional border strip added. The width of the strip is set to half-length of the array A element, so the length of the excitation zone is *L_EZ_* = 38.1 mm and the width *W_EZ_* = 14 mm (Figure 8). The zone is filled with 3456 circular sub-regions of the radius *a* = 0.2 mm. THS method does not allow calculation of normal displacements at the excitation zone, so the line type displacement calculation zone is positioned on the right side of the excitation zone (Figure 8). The length of the displacement calculation zone was set to *L_CZ_* = 60 mm and 0.2 mm step was used.

The pressure signals at the excitation zone and the excited normal displacements of the A_0_ Lamb wave mode at the calculation zone have been calculated for two cases:

(1) All array elements are excited simultaneously.
(2) Array elements are excited one by one with a delay according to Equation (4).

**Figure 10:**
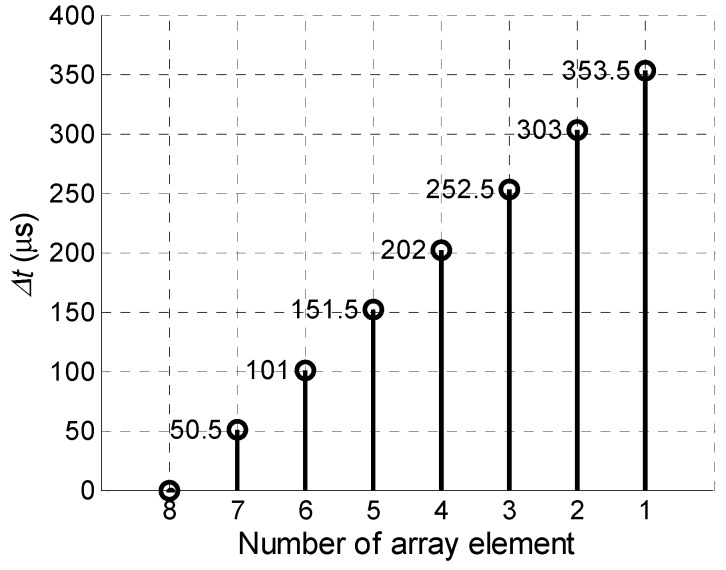
Linear delay scheme of the array excitation signals, when the delay time is increased by *Δt* = 50.5 µs step.

In the second case the delay time for each element has been increased by the step Δ*t* = 50.5 µs, which was calculated according to Equation (4) (Figure 10).

**Figure 11:**
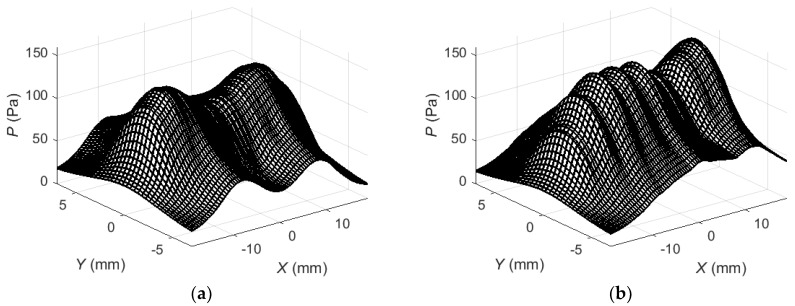
Spatial distribution of acoustic pressure peak values *P* at the excitation zone of the PVC film: (**a**)—simultaneous excitation of all array elements; (**b**)—excitation of array elements with delays.

Then the maximum positive peak values of the obtained acoustic pressure were plotted in Figure 11. In the first case (Figure 11a) no delays for the array elements have been used. In the second case (Figure 11b) a linear delay scheme (Figure 10) of the excitation signals was applied which secures in-phase excitation of the array elements together with a propagating Lamb wave.

As it can be seen from the presented spatial distributions in the case of simultaneous excitation (Figure 11a) there are two clearly expressed peaks. Excitation of the array elements with delays gives complex pressure distribution on the surface of the PVC film (Figure 11b). The acoustic pressure acting on the film creates the Lamb wave propagating in the film.

**Figure 12:**
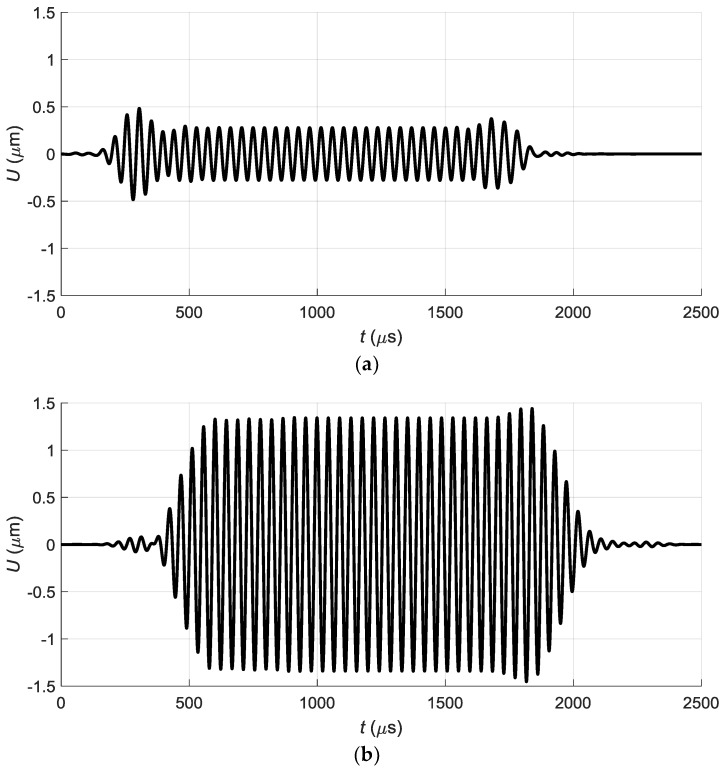
Waveforms of the A_0_ mode normal displacements *U* in PVC film at the point *P_ND_* excited by the linear air-coupled array: (**a**)—simultaneous excitation of all array elements; (**b**)—in-phase excitation of the array elements with delays.

The excited normal displacements of the A_0_ Lamb wave mode on the surface of the PVC film were calculated at the point *P_ND_* (20.1, 0) nearest to the array A and located on *x*-axis (Figure 8). The obtained waveforms of normal displacements for both excitation cases are shown in Figure 12a,b.

**Figure 13:**
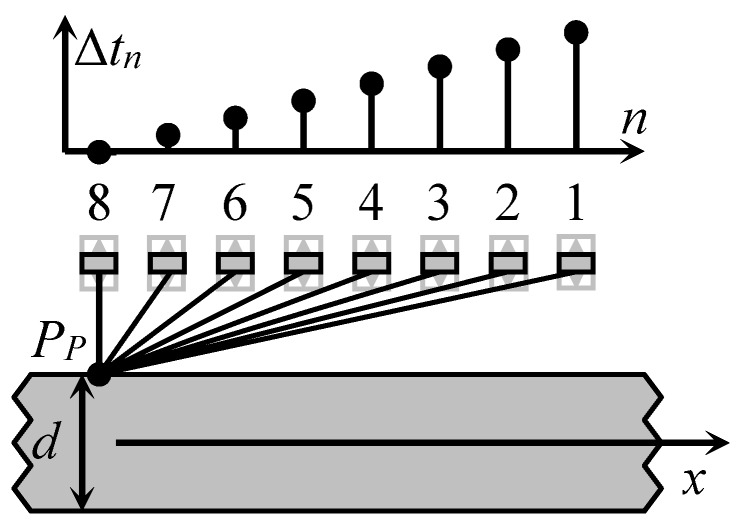
Total acoustic pressure signal at the point *P_P_* as a superposition of partial signals radiated by all 8 array elements. The upper time diagram illustrates the time delays Δ*t_n_* of the excitation signals.

It should be expected that in the case of excitation by linear delayed impulses we should get the biggest amplitude of the off-plane displacements in the film. Actually we see about a 3-fold increase of the amplitude and additional trails in the time domain in comparison with the simultaneous excitation (Figure 12a,b). These additional trails can be explained by the fact that at each point on the surface of the film arrive pressure waves with different delays radiated by all elements of the array (Figure 13). It is like some cross-talk between the array elements taking place via air gap between the array and the film.

**Figure 14:**
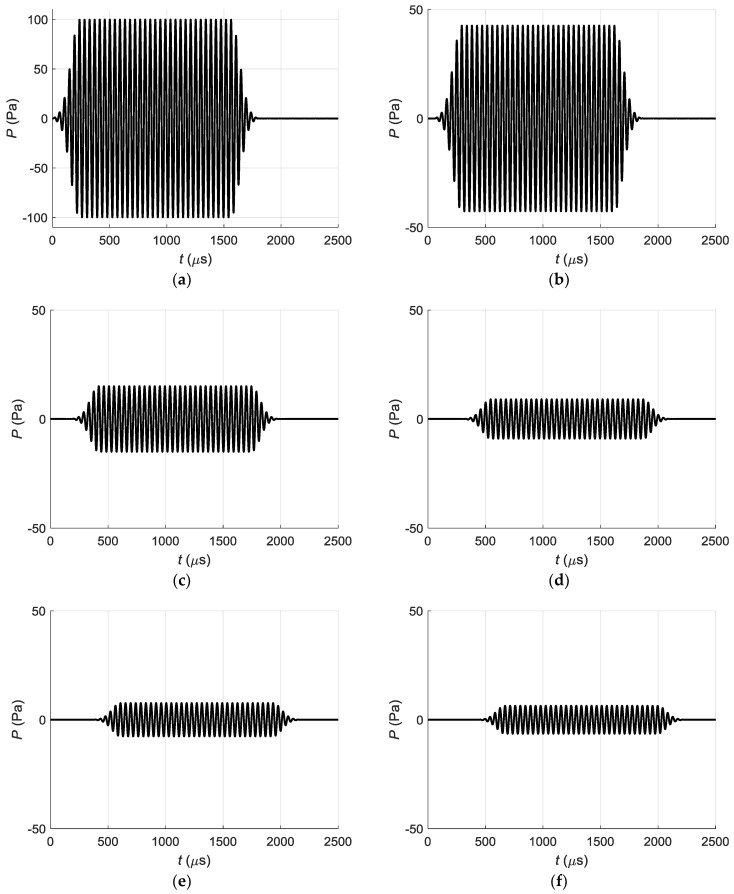
Partial acoustic pressure signals *P* at the point *P_P_*, generated by different array elements: (**a**)—element 8; (**b**)—element 7; (**c**)—element 5; (**d**)—element 3; (**e**)—element 2; (**f**)—element 1.

In order to check this assumption, we have calculated the pressure pulse at the point *P_P_* (Figure 13) when all array elements are excited with the linear delays according to Equation (4). Partial pressure impulses *P* at the point *P_P_*, generated only by different single array elements are shown in Figure 14.

**Figure 15:**
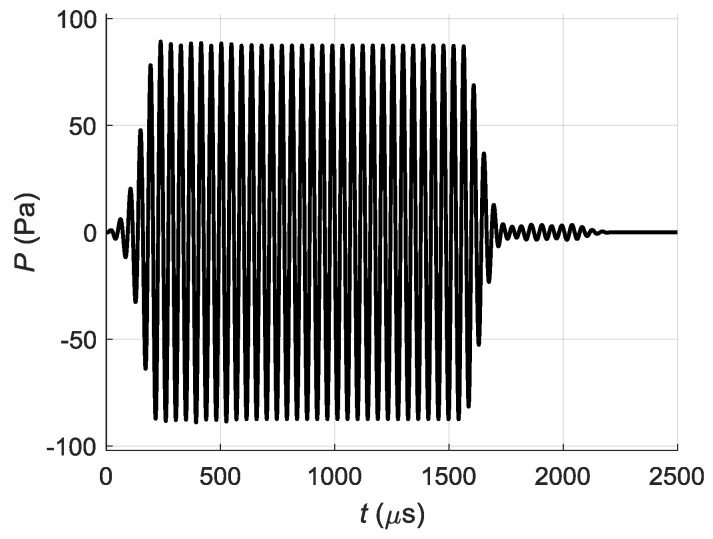
Total acoustic pressure signal *P* at the point *P_P_*.

It is possible to see that as could be expected the impulse with the biggest ≈ 100 Pa maximum amplitude is generated by the element above the point *P_P_*—the element No. 8. The impulses radiated by other array elements are arriving to the point *P_P_* with smaller amplitudes and different delays. The resulting impulse at the point *P_P_* was obtained summing all pulses arriving from all array elements. The obtained in such way pressure impulse is shown in Figure 15.

Its maximum pressure is lower than created only by the element No. 8 and reaches ≈ 90 Pa. Additional trails at the end of the impulse confirms assumption that distortions of the impulse shape and longer duration are caused by cross-talks.

**Figure 16:**
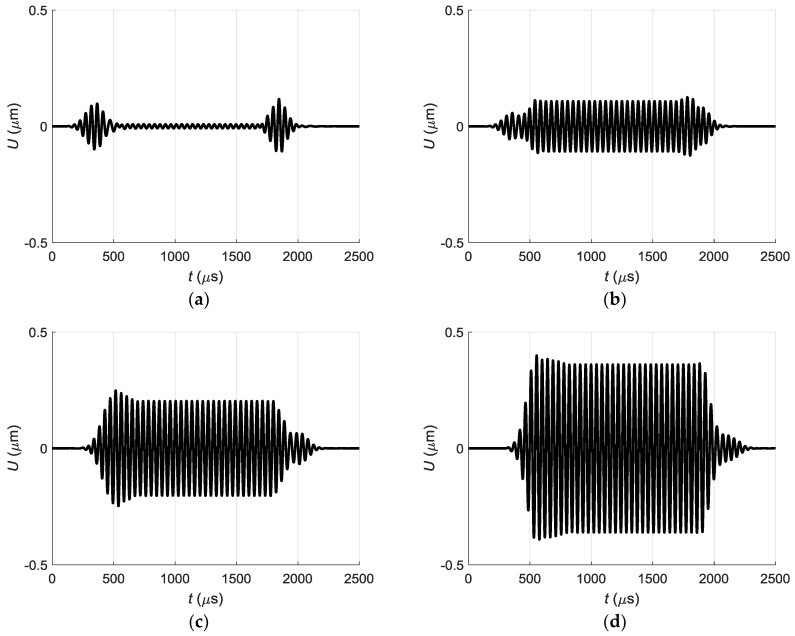

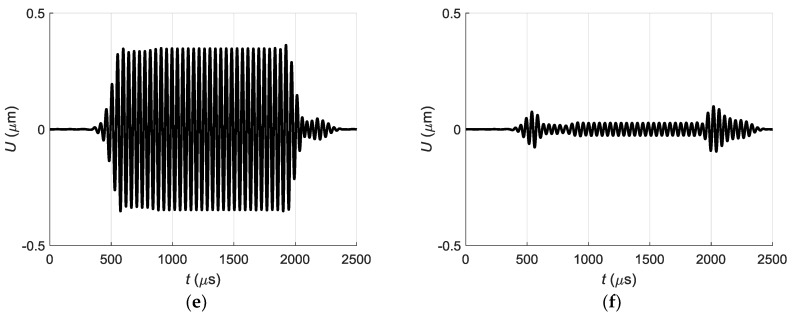
Partial normal displacement signals *U* at the point *P_ND_*, generated by different array elements: (**a**)—element 8; (**b**)—element 7; (**c**)—element 5; (**d**)—element 3; (**e**)—element 2; (**f**)—element 1.

The partial acoustic pressure signals generated by different array elements excite corresponding guided waves in the PVC film which propagate along *x*-axis. They were calculated by the time harmonic solution (THS) method and the obtained displacement pulses at the point *P_ND_* are presented in Figure 16.

If we sum all partial displacement signals radiated by separate array elements we shall obtain the displacement impulse at the point *P_ND_* shown in Figure 12b. This once again confirms the assumption that prolongation of duration and distortions of the shape of the impulse are caused by crosstalk between air-coupled array elements.

**Figure 17:**
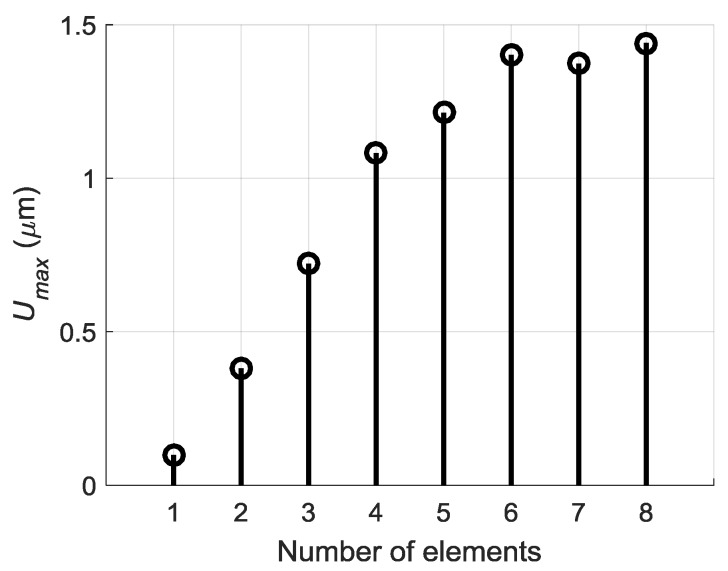
Dependency of maximum displacements amplitude *U_max_* versus number of excited array elements.

Serial excitation of the array elements with the appropriate time delays is based on expectation that the amplitude of a propagating Lamb wave under array aperture will grow due to in-phase excitation of the array elements. In order to check efficiency of such approach we have calculated how the amplitude of the Lamb wave depends on a number of the excited array elements. The results are presented in Figure 17. From the results presented follows that differently from the expected linear growth of the maximal signal amplitude the actual growth is much less. The linear growth is visible for the array elements 1–4. For the elements 5–8 the amplitude growth is not linear, and the amplitude of the total signal obtained from seven elements is lower than the amplitude of the total signal obtained from six elements. It means that superposition process of the normal displacement signals in the film is not optimal and impulses are suppressing each other. This shortcoming may be overcome applying a more sophisticated excitation of the array elements introducing different delay times between excitation instants.

Experimental investigations described in Section 5 showed that pressure signals, radiated by the air-coupled array, propagate also in air and interact with normal displacement signals, which propagate in the PVC film. Propagation velocities of those signals are different—85 m/s for A_0_ mode in the PVC film and 342 m/s in air what means that at some given point *x* those waves arrive with different delays and phases. The acoustic pressure *p*(*x*, *t*) in the wave propagating in air creates also displacements in the PVC film:

*ξ*(*x*, *t*) = *k p*(*x*, *t*) + *u*(*x*, *t*) (6)

where *k* is the conversion coefficient of the acoustic pressure *p*(*x*, *t*) into the normal displacement of the film *ξ*(*x*, *t*). The value of the conversion coefficient *k* was found experimentally by measuring the peak value of the acoustic pressure *p*(*x*, *t*) at the distance 1 mm from the array aperture and the corresponding peak value of the normal displacement *ξ*(*x*, *t*) of the film. The incident pressure *p* with frequency 22.62 kHz was measured by a 1/8” Brüel & Kjær microphone (B&K 4138-A-015, Brüel & Kjær, Naerum, Danmark). The excited displacement field in the film was measured by the Polytec laser interferometer OFV-5000. The measured value was *k* = 2.5 × 10^−8^ m/Pa.

**Figure 18:**
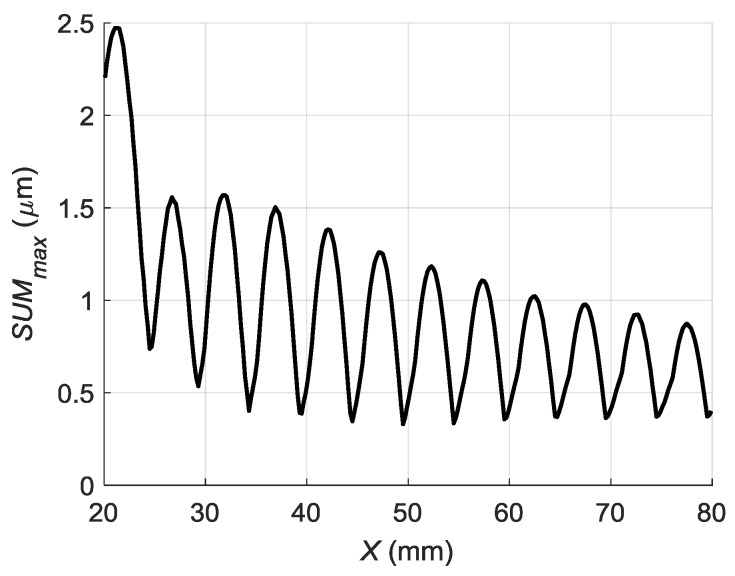
Spatial distribution of the peak values of the normal displacements of the PVC film along *x*-axis taking into account the wave propagating in air.

Spatial distribution of the total normal displacement amplitudes *ξ*(*x*) along *x*-axis calculated using Equation (6) and the measured conversion coefficient *k* is presented in Figure 18.

From the simulation it follows that the normal displacement amplitudes instead of monotonous decay along *x*-axis due to diffraction and losses undergo periodic oscillations caused by interference of the guided wave and the wave propagating in air. As it will be shown in Section 6 experiments confirm this phenomenon.

## 4. Optimization of the Excitation Process

It was presumed that optimisation of normal displacements signals can be performed by applying the additional time shifts to the excitation signals of the array elements. The necessary time shifts *t_n__opt_* for each array element were found looking for the time delay *t_n_*_+1 *opt*_ at which the recursive function $\xi_{n}^{r}\left(t\right)$ reaches the maximum:

(7) $$t_{n+1opt}=argmaxt_{n}\left[\xi_{n}^{r}\left(t\right)+\xi_{n+1}\left(t-n\Delta t-t_{n+1}\right)\right],$$

(8) $$\xi_{n+1}^{r}\left(t\right)=\xi_{n}^{r}\left(t\right)+\xi_{n+1}\left(t-n\Delta t-t_{n+1opt}\right),$$

where $\xi_{n}^{r}\left(t\right)$ is the resultant off-plane displacement at the point *P_ND_* depending on the number *n* of the excited array elements (*n* = 1, 2, …, *N*), ∆*t* is the delay time required by the linear delay approach (Equation (4)) and *t_n_*_+1_ is the additional delay time for the *n*’th array element the optimal value of which *t_n_*_+1_ opt must be found. The optimization process is performed in a recursive way: step-by-step procedure which is repeated (*N* − 1) times, where *N* is the number of the array elements. During the first step the displacement *ξ*_1_(*t*) created only by the first array element (No. 8) is taken as the reference signal. Then the second element after delay ∆*t* is also excited and the displacement *ξ*_2_(*t* − ∆*t*) is used as the second term in Equation (7). After that shifting of the signal *ξ*_2_(*t* − ∆*t* − *t_n_*_+1_) in the time domain is performed until a maximal value of the resulting signal $\xi_{2}^{r}\left(t\right)$ is achieved and the optimal delay time *t_n_*_+1_ opt for excitation of the second array element is found.

After that during the second step the obtained resulting signal $\xi_{2}^{r}\left(t\right)$ is put into the right side of Equation (7) as the displacement signal *ξ*_2_ and all procedure is repeated once again in the same way—the signal *ξ*_3_(*t*) excited by the third array element is taken, all calculations are repeated, etc. The recursive optimization procedure is finished when all array elements are excited and all additional correction times for all elements are found.

In the previous section it was shown that the normal displacement of the film *ξ*(*x*, *t*) at the point *x* = *P_ND_* is caused by interference of the A_0_ mode and the wave propagating in air. This interaction must be taken into account during optimisation. In this case the displacement signal given by Equation (6) must be used during optimization procedure described by Equations (7) and (8).

**Figure 19:**
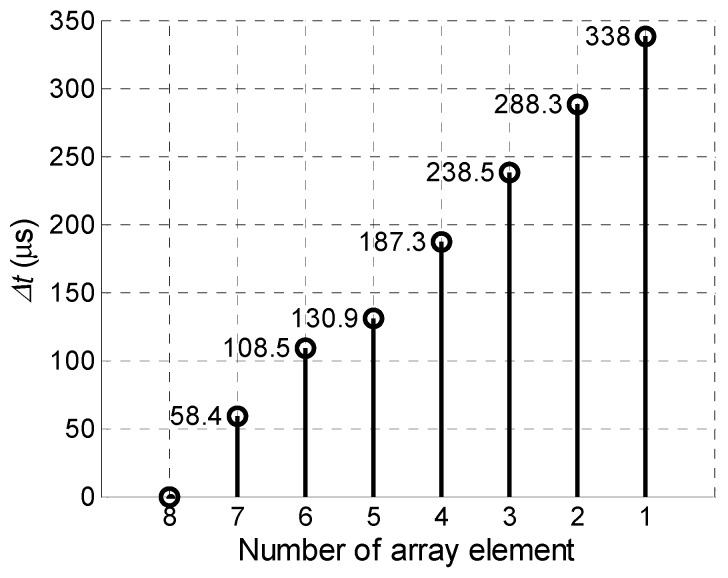
Optimised delay scheme of the array excitation signals.

The obtained in such way delay scheme of the excitation signals is presented in Figure 19.

**Figure 20:**
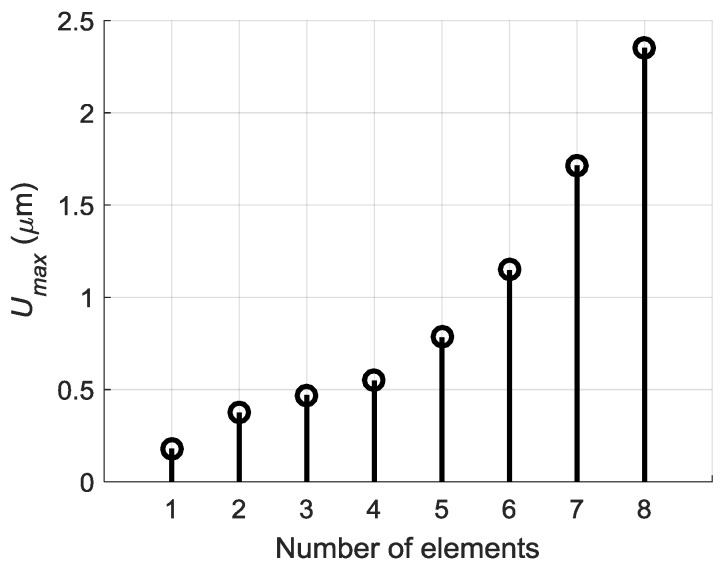
Dependency of peak values of normal displacements *U_max_* of the PVC film at the point *P_ND_* versus number of the excited elements after applying advance times.

The maximal amplitudes of off-plane displacements observed after such procedure at the point *P_ND_* on the surface of the PVC film versus the number excited array elements are shown in Figure 20. If to compare Figure 20 with data presented in Figure 17 we can see a significant improvement—after optimization procedure the amplitude grows progressively with the number of excited elements.

**Figure 21:**
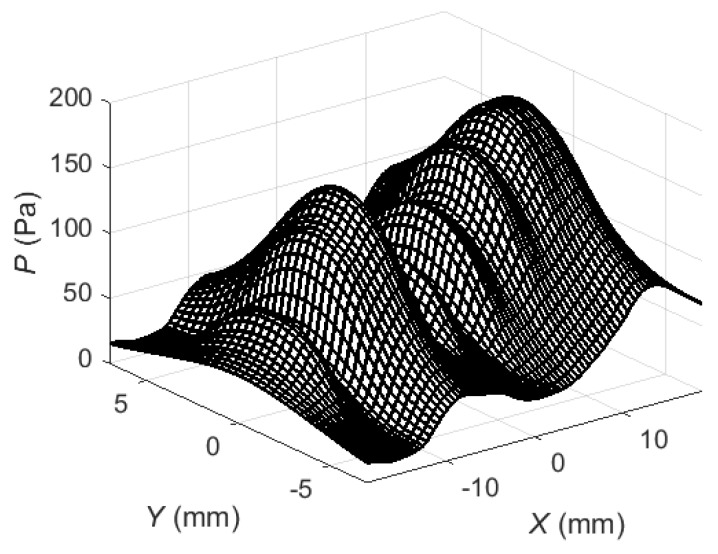
Spatial distribution of the maximum positive peak values of the acoustic pressure at the excitation zone when the corrected time delays between array elements were used.

**Figure 22:**
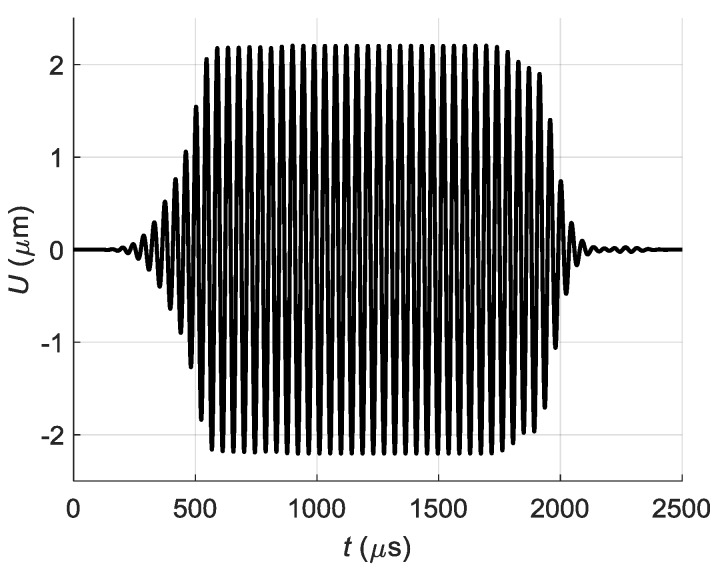
Impulse of normal displacement of the PVC film at the point *P_ND_* (Figure 8) when the corrected time delays between array elements are used.

The entire excitation process has been modelled using the new stepped delay scheme and then maximum positive peak values of the acoustic pressure (Figure 21) and the maximum positive peak values of the normal displacements of the PVC film (Figure 22) were calculated and plotted.

Application of the corrected time delay scheme of the excitation instants of the array elements changes essentially the spatial distribution of the acoustic pressure at the excitation zone under the air-coupled array (Figure 21): the maximum values are concentrated at the side next to the calculation zone on the PVC film and are about 1.2 times bigger than in the linear delay case (Figure 11b). The spatial distribution of normal displacements of the PVC film (Figure 22) shows an increase of the peak values of about 1.6 times in comparison with the linear delay case. The impulse of normal displacements at the point *P_ND_* (Figure 22) shows almost 1.6 times higher amplitude and minimized trails at the beginning and the end of the impulse. Please note that the corrected excitation time instants may be calculated once before and used after that in all experiments.

## 5. Experimental Investigations

**Figure 23:**
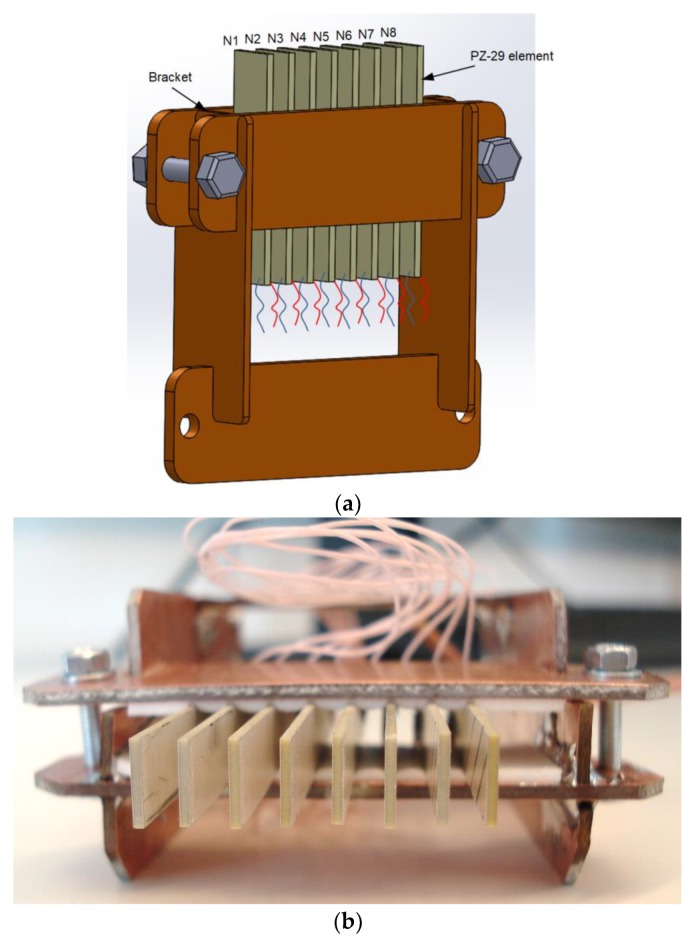
The prototype of the air-coupled 8 element array: (**a**)—3D view; (**b**)—top view.

In order to check possibility to excite a slow Lamb wave in a thin PVC film with the proposed air-coupled ultrasonic array experiments were performed with the 8 element array. The array was assembled of rectangular piezoelectric strips with dimensions (60 mm × 7 mm × 1 mm) vibrating in a transverse length mode (Figure 23).

The array frame was made of 1.6 mm thickness double-sided copper clad laminate board FR4 (Shenzhen Core-Tex Composite Materials Co. Ltd., Shenzhen, China), and the piezoelectric elements have been fixed to the frame using double-sided bonding tape.

**Figure 24:**
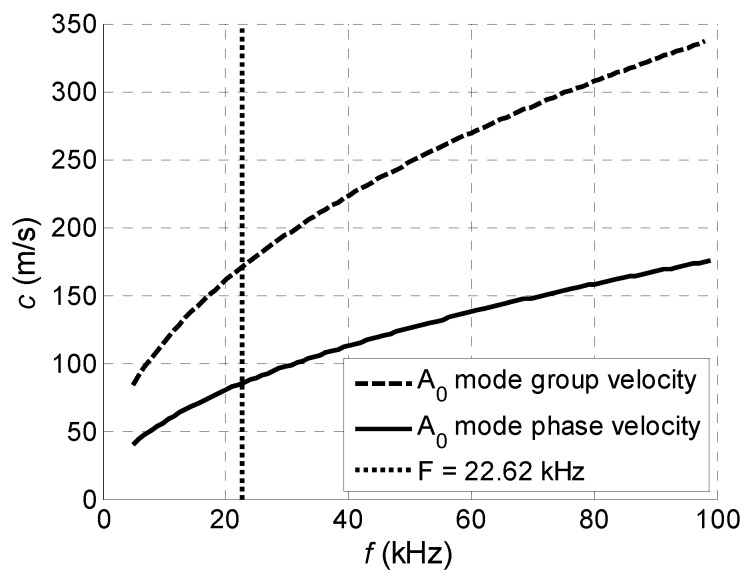
The calculated phase and group velocity dispersion curves for *d* = 0.13 mm thickness clear PVC film.

For radiation into air the tips of the piezo ceramic strips with a rectangular aperture (7 mm × 1 mm) were exploited. The piezoelectric strips used as array elements were manufactured of piezo ceramics Pz-29 (Meggitt A/S, Kvistgaard, Denmark). The electromechanical coupling factor for a transverse length mode *k*_31_ = 0.37 is the highest among all piezo ceramics materials provided by Ferroperm™ Piezoceramics. The main length resonance frequency of the piezoelectric strips was *f* = 23.3 kHz. However, Lamb wave excitation experiments in PVC film showed, that the maximum amplitude of normal displacements has been achieved when a slightly lower frequency *f* = 22.62 kHz was used. For that reason, this frequency was used in numerical simulations. Therefore, to select properly the pitch between elements which should be equal to the wavelength of the A_0_ mode, the dispersion curves for this mode were calculated (Figure 24). At the selected frequency *f* = 22.62 kHz the phase velocity in the PVC film is *c_ph_* = 85.1 m/s, and the wavelength of the A_0_ mode λ*_A_*_0_ = 3.8 mm. The nominal pitch between the manufactured array elements was designed to be 4.3 mm, but the real pitches are of slightly different sizes, as it was mentioned in Section 3. However, as it was mentioned in Section 2, variances of the pitches can be compensated by selecting suitable delays of the excitation signals.

**Figure 25:**
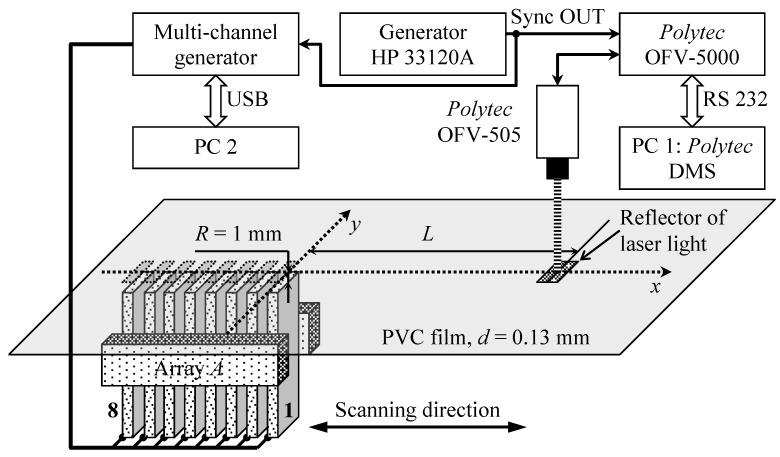
Experimental set-up used for air-coupled excitation of a slow A_0_ Lamb wave.

The experimental set-up used for excitation and reception of slow A_0_ mode Lamb wave is shown in Figure 25.

The array was fixed to software controlled XY scanner 8MTF-75LS05 (Standa Ltd., Vilnius, Lithuania). The PVC sample was A4 size—210 × 297 mm and with the thickness *d* = 0.13 mm. This sample was fastened in a stationary frame at the *R* = 1 mm distance from the array. The ultrasonic array was excited by the multichannel signal generator SITAU 32:128:2 LF TR (DASEL Systems, Madrid, Spain) which was supplying to each array element the 22.62 kHz square impulses with the duration 40 periods and the 60 V amplitude, which were delayed according to different schemes. The ultrasonic wave radiated through the air gap excites in the clear PVC film the slow A_0_ Lamb wave which creates off-plane displacements in the film. Those displacements were registered by the Polytec laser interferometer OFV-5000 (Polytec GmbH, Waldbronn, Germany). In order to improve reflection of the laser beam, a small square 1 × 1 mm reflector was glued on the surface of the clear PVC film (Figure 25). The HP 33120A generator (Hewlett-Packard, Palo Alto, CA, USA) acts as a synchronization unit between ultrasonic and optical measurement systems.

**Figure 26:**
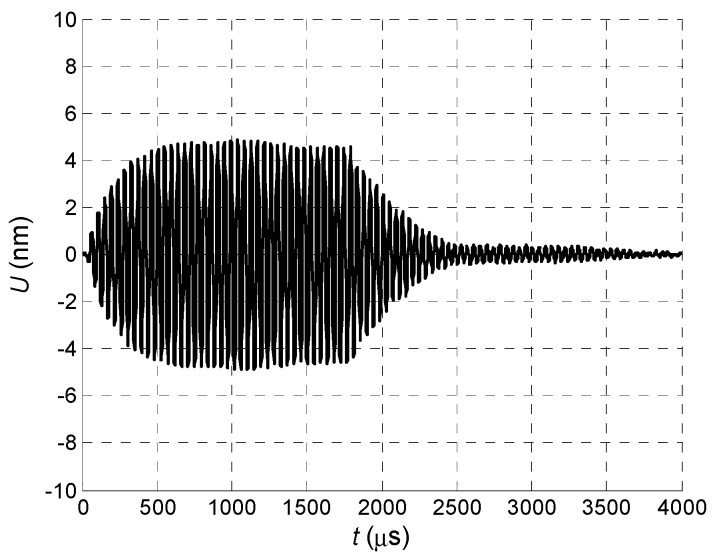
Normal displacement signal *U* of the clear PVC film, measured at *L* = 1 mm, when all array elements are excited simultaneously.

The normal displacement impulses were recorded by the Polytec laser interferometer at the distance *L* = 1 mm from the air-coupled array element No. 1 in three cases. In the first case all array elements were excited simultaneously (Figure 26).

**Figure 27:**
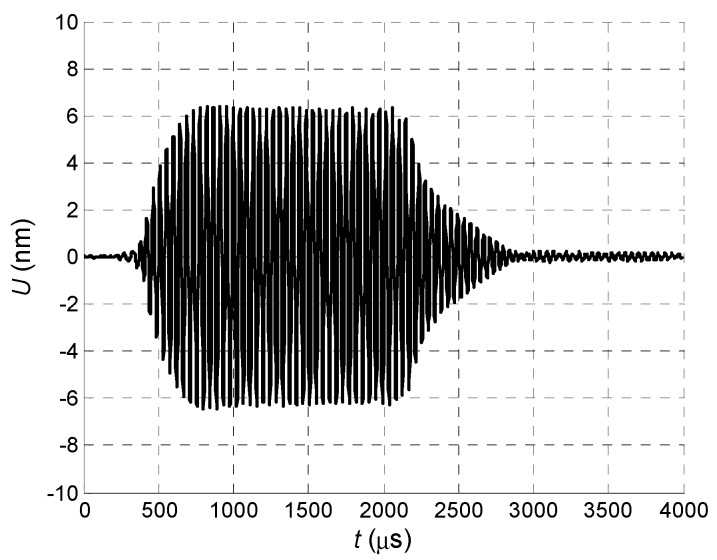
Normal displacement signal *U* of the clear PVC film, measured at *L* = 1 mm, when all array elements are excited using delay scheme with equal *Δt* = 50.4 µs step.

In the second case all array elements were excited using the delay scheme with the constant steps Δ*t* = 50.4 µs (Figure 10) and the obtained waveform is shown in Figure 27.

**Figure 28:**
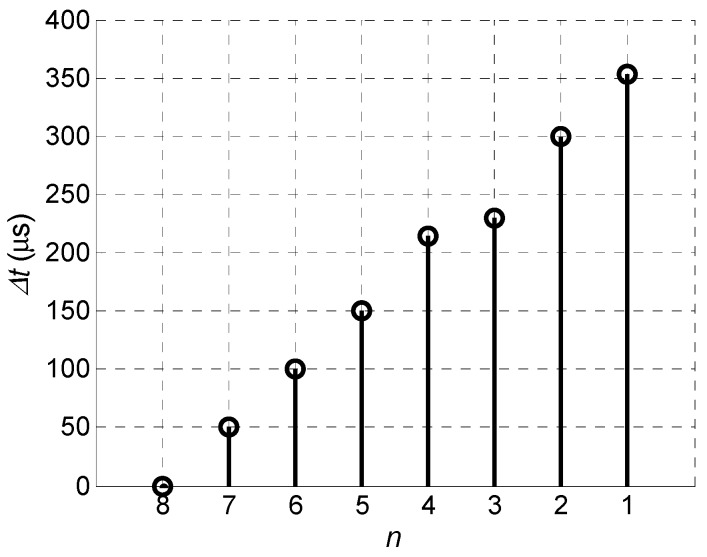
Experimentally obtained optimised delay scheme.

**Figure 29:**
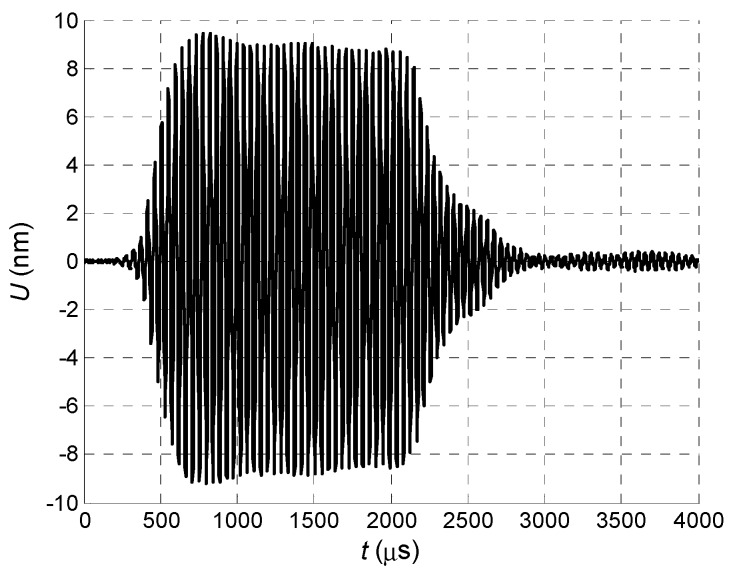
Normal displacement signal *U* of the clear PVC film, measured at *L* = 1 mm, when all array elements are excited using optimized delay scheme.

In the third case the optimised delay scheme of the excitation pulses was used. The necessary delay times were found using the before described optimization algorithm—delay time for each element has been slightly increased and decreased step-by-step searching for maximum of the normal displacement signal at the distance *L* = 1 mm from the first array element (Figure 28 and Figure 29).

**Figure 30:**
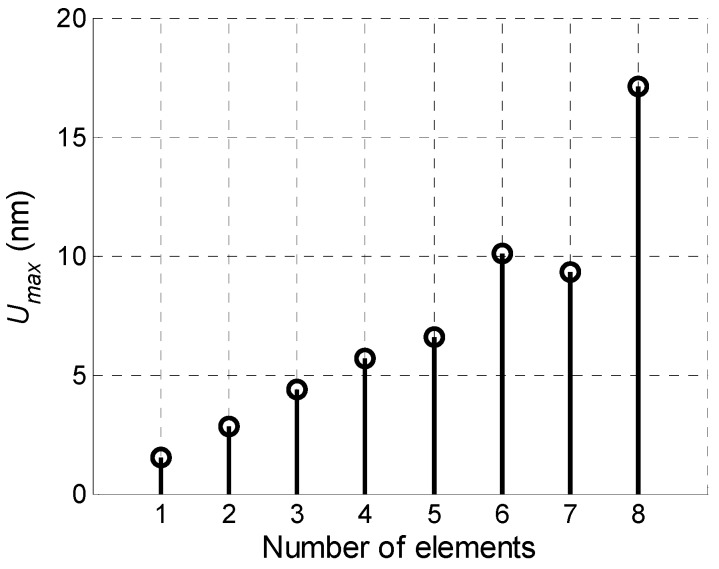
Maximal amplitudes of the normal displacement signal *U_max_* of the PVC film, measured at *L* = 1 mm versus number of the excited array elements in the case of the optimised delay times.

Comparison of the measured displacement signals shows that the best result is obtained in the case of the optimised delay scheme—the normal displacement signal in this case is 1.5 times higher than using the delay scheme with a constant step and 2 times higher when all array elements are excited simultaneously. Dependency of the measured maximal signal amplitude *U_max_* versus number of the excited array elements in the case of the optimised delay scheme is shown in Figure 30.

From the presented results it follows that this in the case of excitation of all eight elements is 11.4 times bigger than in the case of one element. It is very close to the simulation result which is 12 times (Figure 19).

**Figure 31:**
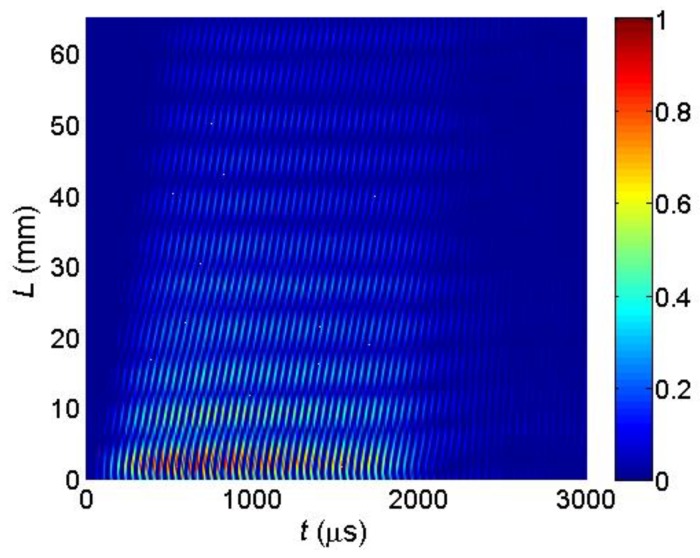
Measured B-Scan of the normal displacements of the PVC film when all array elements were excited simultaneously.

**Figure 32:**
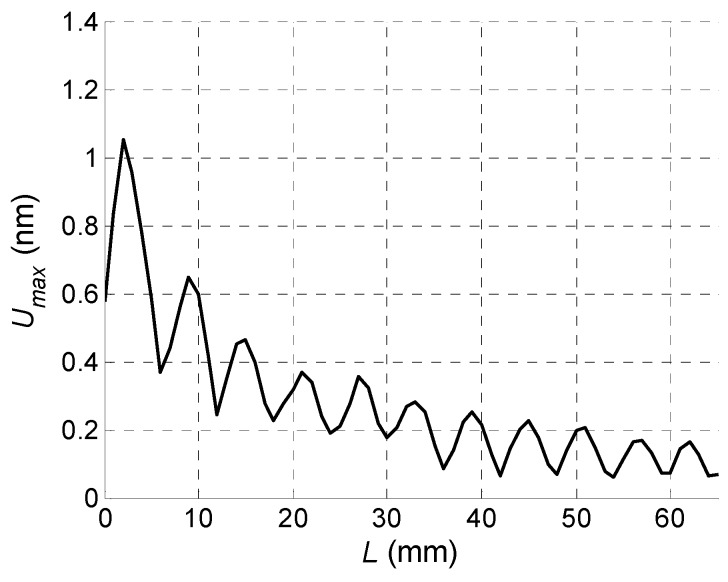
Spatial distribution of the peak values of the normal displacements *U_max_* of the PVC film when all array elements were excited simultaneously.

The key question is if the normal displacements measured by the laser interferometer are caused by a desirable A_0_ mode Lamb wave or not. This question can be answered by measuring the propagation velocity of the picked up normal displacement pulse and comparing it to the velocity of the A_0_ mode following from the dispersion curve (Figure 6). However, this task is complicated by the fact that total normal displacement is caused not only by the guided wave propagating in the film, but also by a direct wave in air which is much faster than the guided wave. It follows from the B-Scans measured by the laser interferometer along *x*-axis (Figure 25). In the first case the B-Scan (Figure 31) was recorded when all array elements were excited simultaneously and dependency of the maximum amplitude along *x*-axis was plotted (Figure 32).

**Figure 33:**
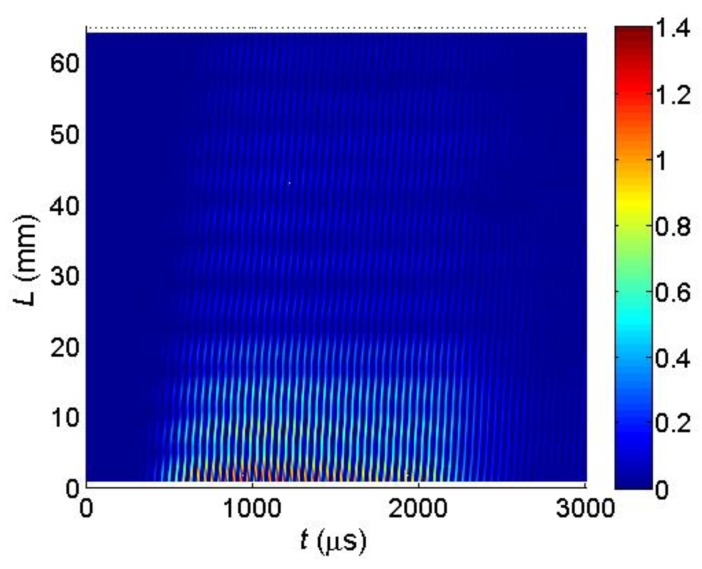
Measured B-Scan of the normal displacements of the PVC film when the optimised time delays between array elements were used.

**Figure 34:**
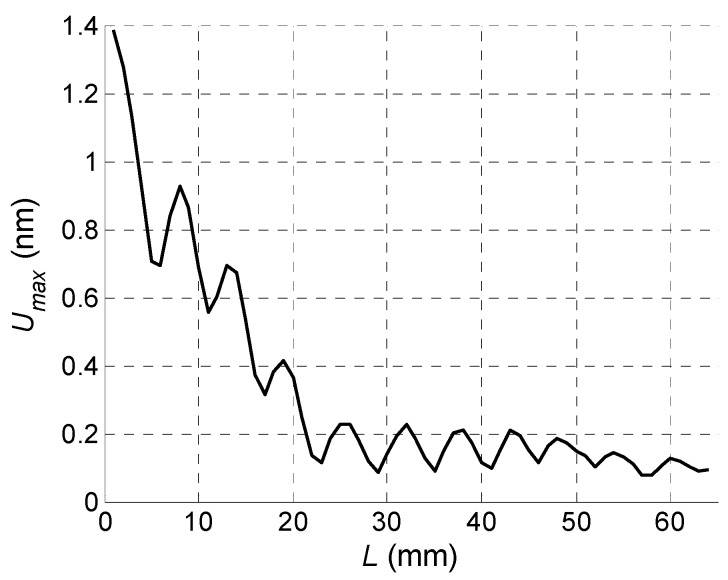
Spatial distribution of the peak values of the normal displacements *U_max_* of the PVC film when the optimised time delays between array elements were used.

In the second case the B-Scan (Figure 33) was recorded when all array elements were excited using the experimentally obtained optimised delay scheme (Figure 28), and the dependency of the maximum amplitude was plotted (Figure 34).

From the presented results strong periodic variations of the signal amplitude along *x*-axis are observed. Those variations are caused by interference of the A_0_ mode and wave propagating in air. Measurements of the ultrasound velocity using such interfering signals may be quite inaccurate. Therefore, for solution of this problem filtering of the measured normal displacement signals was performed. For this purpose, we have used 3D filtering of experimentally measured C-Scan in a spatial-temporal domain proposed in [29]. The ultrasound velocity measured after filtering was 89 m/s, which is rather close to the velocity of the A_0_ mode *c_ph_* = 85.1 m/s at the frequency *f* = 22.62 kHz predicted from the dispersion curve shown in Figure 24.

## 6. Conclusions

In thin plates and flexible films like clear PVC films guided Lamb wave A_0_ mode at lower frequencies may propagate with a velocity lower than the ultrasound velocity in air. This velocity is frequency dependent and is decreasing with a decreasing frequency. In this case according to the Snell’s law air-coupled excitation of such mode seems to be impossible. This problem can be solved by applying for excitation a multi-element linear air-coupled array. The distance between elements of the array is matched to the wavelength of the A_0_ mode in a film.

The elements of the array may be excited simultaneously or one by one with a delay corresponding to the propagation time of the A_0_ mode in the film between neighbouring elements of the array. Both methods enable air-coupled excitation of the A_0_ mode in the film. The second method should be most efficient, but the performed simulations revealed that in the air gap between the array and the film crosstalk between array elements takes place, which reduces the excitation efficiency and distorts waveforms of the impulses in the film. In order to overcome this shortcoming, we have proposed the optimization procedure based on additional shifts of electric excitation impulses of the array elements. This procedure enables to increase almost two times the amplitude of excited ultrasonic impulses and at the same time to reduce distortions of the waveforms caused by cross-talks.

For experimental verification of the proposed approach a prototype of the air-coupled eight element array made of Pz-29 piezoceramic strips was manufactured. The off-plane displacements of the PVC film were recorded by the Polytec OFV-5000 laser interferometer. Experimental validation confirmed possibility to excite the slow A_0_ Lamb wave mode through air gap in thin plates and films.

It was shown that by selecting delay times of the excitation pulses of different array elements it is possible to excite the waves the wavelength of which is not strictly equal to the pitch between elements. It means that variation of the delay times allows adapting such array to films and thin sheets with different ultrasound velocities.

## Figures and Tables

**Table 1:** Parameters of clear PVC film.

Parameter	Value
Density	*ρ* = 1400 kg/m^3^
Young’s modulus	*E* = 2156 MPa
Poisson’s coefficient	*ν* = 0.42

**Table 2:** Coordinates of array A element centres.

El. No.	1	2	3	4	5	6	7	8
*X_T_*, mm	15.05	10.75	6.45	2.15	−2.15	−6.45	−10.75	−15.05
*X_R_*, mm	14.75	10.65	6.45	2.15	−2.15	−6.45	−10.75	−14.55
